# An Optimized Framework for WSN Routing in the Context of Industry 4.0

**DOI:** 10.3390/s21196474

**Published:** 2021-09-28

**Authors:** Shalli Rani, Deepika Koundal, Kavita, Muhammad Fazal Ijaz, Mohamed Elhoseny, Mohammed I. Alghamdi

**Affiliations:** 1 Chitkara University Institute of Engineering and Technology, Chitkara University, Punjab 140401, India; 2 Department of Systemics, School of Computer Science, University of Petroleum and Energy Studies, Dehradun 248001, India; koundal@gmail.com; 3 Department of Computer Science and Engineering, Chandigarh University, Mohali 140413, India; kavita@ieee.org; 4 Department of Intelligent Mechatronics Engineering, Sejong University, Seoul 05006, Korea; fazal@sejong.ac.kr; 5 Faculty of Computers and Information, Mansoura University, Mansoura 35516, Egypt; melhoseny@ieee.org; 6 Department of Computer Science, Al-Baha University, Al-Baha City 1988, Saudi Arabia; alghamdi@alumni.nmt.edu

**Keywords:** OLSR, Industry 4.0, Smart Grid (SGs), routing, MANET, ad hoc network

## Abstract

The advancements in Industry 4.0 have opened up new ways for the structural deployment of Smart Grids (SGs) to face the endlessly rising challenges of the 21st century. SGs for Industry 4.0 can be better managed by optimized routing techniques. In Mobile Ad hoc Networks (MANETs), the topology is not fixed and can be encountered by interference, mobility of nodes, propagation of multi-paths, and path loss. To extenuate these concerns for SGs, in this paper, we have presented a new version of the standard Optimized Link State Routing (OLSR) protocol for SGs to improve the management of control intervals that enhance the efficiency of the standard OLSR protocol without affecting its reliability. The adapted fault tolerant approach makes the proposed protocol more reliable for industrial applications. The process of grouping of nodes supports managing the total network cost by reducing severe flooding and evaluating an optimized head of clusters. The head of the unit is nominated according to the first defined expectation factor. With a sequence of rigorous performance evaluations under simulation parameters, the simulation results show that the proposed version of OLSR has proliferated Quality of Service (QoS) metrics when it is compared against the state-of-the-art-based conventional protocols, namely, standard OLSR, DSDV, AOMDV and hybrid routing technique.

## 1. Introduction

The fourth stage of the industrial revolution, i.e., Industry 4.0, has increased its rate of growth due to the development of Information and Communication Technologies (ICT) [1]. Industry 4.0 has served as the base of intelligent processes by controlling and enhancing the production processes by using the new techniques of ICT. Moreover, inter-operability among the products, machines, operators, and spare parts has created a demand for reliable and stable connectivity with the autonomous interaction among the various subsystems and systems in Industry 4.0. IoT is proving itself as the best way to connect real and virtual worlds, which is a total game-changer and revolution for Industry 4.0 networking and development. Both types of communications are possible with IoT, i.e., wired and wireless. The main aim of the communication layer is to facilitate the automated exchange of data in smart industries [2]. The design and implementation of industry framework in stable networking for heterogeneous systems are very challenging due to the diversified requirement of QoS for smart industry applications [3]. Therefore, it is very crucial to understand the need for QoS suite/metrics in industrial processes for deploying the communication framework of Industry 4.0 [4]. The Quality-of-Service (QoS) attentive cooperative communication in IoT applications such as Smart Grids (SGs) is reliant on various performance measures/metrics such as time, stability, network lifetime, and complexity.

In SGs, a broad range of Mobile Ad hoc Network (MANET) mechanisms have been presented in the literature so far. However, intelligent SGs Industry networks cannot be facilitated by traditional protocols of MANET. They make data communication a challenging task for SGs applications. Under hostile environments, quality-aware metrics such as latency, network lifetime, reliability, and complexity are the major issues of SGs. Hence, the process of optimization is required in traditional communication protocols of MANET for SGs Industry 4.0. The connectivity between nodes in MANET is processed directly without any interference of fixed equipment in the particular mobility administration of MANET.

Optimized Link State Routing (OLSR) is considered as an effective MANET protocol, which is used for ensuring a destination track in a routing list [5].

We examined two protocols, Ad hoc On-Demand Distance Vector (AODV) [6] as a responsive show and OLSR as a proactive show for Industry networks.

To deal with above-mentioned challenges, this paper proposes an improved OLSR protocol for SGs in MANET to improve the management of control intervals that enhances the efficiency of the standard OLSR protocol without affecting its reliability. The adopted process of grouping reduces the total energy costs by reducing severe flooding in evaluating a head of clusters and has improved the communication services in SGs.

### Problem Definition and Motivation

The prime motive of the SG Industry 4.0 is to offer a smart electricity paradigm by use of innovative IoT technologies to provide various benefits in the emerging fields: industry economy, constrained sources of energy, reliability, network stability, security, etc. [7]. In SG Industry 4.0, the subsystems and components of the framework will be inter-operable and collaborate strictly. This focuses on the optimizations in the existing frameworks of MANET. One of the famous and existing approaches forms a MANET among the sensor nodes, which is based on the controlling of messages in wireless communication is OLSR that is suitable only for SGs traditional paradigms. However, OLSR in RFC 3626 confirmed that control nodes, if productive for a particular procedure, send control messages at different stages [8]. The above discussion of Industry 4.0 constraints and use of MANETs for industrial applications has motivated us to propose a new protocol that focuses on the management of interval of control messages, which helps in robust communication in SG Industry 4.0.

The proposed approach would provide solutions to issues discussed in the previous subsection by improving the management of control levels in ad hoc sensor networks. This paper has the following contributions:

1. We propose an optimized routing scheme by updating control interval levels, which reduces the total network cost by lessening the severe flooding and evaluating a head of clusters on the basis of the probability discussed in the proceeding sections. Delay due to control intervals is not tolerable in communication paradigms of SGs, which is solved by the proposed methodology.
2. The novel scheme is based on the selection of the head of the unit responsible for control messages according to the first defined expectation factor.
3. We also selected the optimal path based on multiple parameters such as the mobility of nodes, network scenario as per the connectivity of nodes, energy level of sensor nodes, and missing links between the nodes.
4. With a sequence of rigorous performance evaluations under QoS parameters, the analysis demonstrates that an optimized version of OLSR (OCIR) has improved the QoS metrics. The work ensures the reliable communication for SG applications with low overhead.

The rest of the paper is organized as follows: Section 2 shows the highlights of the recent literature of SGs and MANET followed by the proposed work in Section 3. Section 4 presents the result analysis and discussions. Finally, the paper is concluded in Section 5.

## 2. Related Work

A recent research survey reveals that a lot of work is being carried out on Industry 4.0 in terms of new frameworks, protocols and algorithms. MANETs in various regions produce numerous mobile nodes and those are free to communicate. In OLSR, Multi-Point Relays (MPRs) are single nodes that are preferred to buy compared to special edition nodes that can be susceptible to MPR nodes for a huge energy guideline. On the downside, MPR nodes that compete excessively help the resources by dropping numerous node packets in preference to sending them. This leads to enormous energy loss and vitiates execution in certain current energy-efficient MPR choice initiatives. For this service, MANET is instituted by an upgraded energy and stability-conscious routing model [9,10]. Pourghebleh et al. [11] invented methodology that states each IoT machine has a peculiar ability to spare its detection and sensing of information or data. It is capable of receiving information from the other devices. The algorithms proposed have the primary goal to combine existing data and to improve the proficiency of the following such as traffic bottleneck, consumption of energy, increase the IoT lifetime, and the amount of traffic injected into the network. In [12], the authors proposed an energy efficient network; however, it is not based on multiple parameters. Some authors proposed [13] secure communication for data transmission, which is suitable only for the social activities rather than industrial processes. The QoS-based approach is followed in [14]; however, smart grid applications require a main focus on control packets and reliability, which is why some optimizations are required. Unless with the help of network slicing [15,16], this cannot be gained. As per the literature, augmented reality systems [17] are also focusing on the industry perspectives; however, an integrated mechanism is required to deal with industrial constraints.

The authors of [18,19] presented a lifetime data balanced mechanism on the IoT, which is run by an end-to-end delay requirement to manage the system framework dynamics and heterogeneity while trying to improve the energy level of the nodes for IoT applications. The main challenges of the smart grids are not solved by the proposed methodologies. The authors described and presented the OLSR technique’s control strategy in [20,21]. There are many smart applications of WSN and where the researchers have worked upon QoS metrics. However, the authors of [22] proposed a routing framework for smart grid application in the context of Industry 4.0. They have worked on end to end reliability. Other technologies are also paving the way for Industry 4.0 such as LoRaWAN [23]. Authors have also worked on the energy emission of motes. Cyber physical integration is proposed in [24] for smart ship manufacturing.

The proposed methodology of this paper, i.e., the multipoint relays (MPR) technique, is used to optimize the flow created by messages used in the discovery of the neighborhood, and it is the main requirement of SGs, which is idealistic for industry 4.0. The presented approach is based on the routing approach proposed in [22]. The present article has introduced the reduction of time between the control messages and hence also reduced the delay. In SG applications, delay, optimal path for transmitting the data, and missing link identification are prime requirements, which are covered, analyzed and evaluated by the proposed methodology over OLSR, hybrid routing, AoMDV and DSDV [25] protocols.

## 3. Proposed Framework

The OLSR performance relies upon the duration of the interval of *hello* messages and neighbor’s hold time. Control messages, i.e., are the *hello* messages’ notice modification in associations of neighbor nodes. In the case of a missing link, the data and control packets are not transmitted to the missing route. The neighbor’s hold time is an extreme waiting period for which a node waits for a link to reply to a symmetric link in the case of a missing route. A new shortest route is selected to transmit packets and to avoid the missed link data transmission. The default interval of hello messages is two seconds in OLSR and neighbor’s holding time is (3×hello interval (2 s)), i.e., 6 s [20]. The terms used in mathematical analysis are given in Table 1.

**Figure 1:**
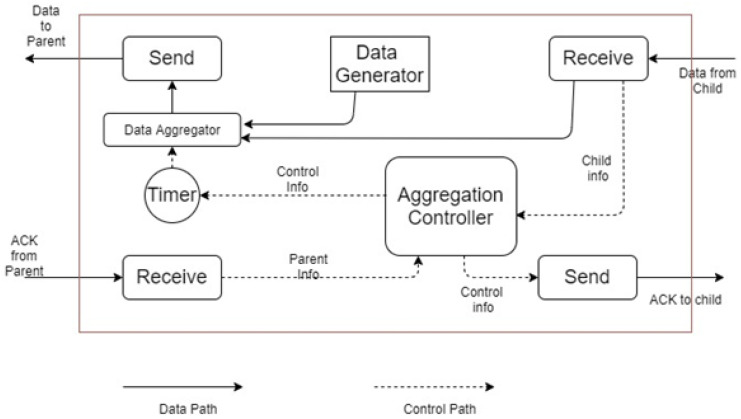
Design overview of the routing scheme.

The whole working steps of the above discussion are shown in Figure 1 and demonstrated in the subsequent sections. The following steps are used to set up the environment for the optimized OLSR for SGs Industry 4.0, and the request transmission can be observed from Algorithm 1.
**Algorithm 1:** Resource Request Forwarding**Input**: SourceN, DestN, RIdusreq, Set of RNs, and channel_info_table**Output**: Route_req_info transmission**Begin:**1   if SourceN_route to DestN!=valid_route or SCFT(selected channel2   for fixed duration) == expired then3   SourceN creates route_req_pkt (RRP) with new id4   Add id, RIdusreq and channel_info_table to RRP5   single hop neighbor_node (NN) member of SourceN and Adj_list>06     transmit route_req_pkt to all channels7   **end_for**8   **end_if**9   for each rn in RNs10   if info_received= Route_req_info11              If (info_received==info_received from previous channel or12               Route_req_length>=previous Route_req_info then13               Discard info_received14              end_if15   else16   Compute $T1_{rn}^{Flow\_ser}$(t1) as per the Equation (18)17   Add id, $T1_{rn}^{Flow\_ser}$(t1), channel_info_table and Route_req_info18   single hop neighbor_node (NN) member of RN with Adj_list>019              broadcast Route_req_info to all channels20   **end_for**21   **end_else**22   **end_if**23   **end_for****End**

### 3.1. Initial Premises

In order to provide the mathematical analysis of OLSR and AODV configuration parameters, we determined three initial premises that serve us with a better description of the behavior of these routing protocols: Maximal speed of node’s movement: s = 10 ms${}^{-1}$; transmitting range with radius: r = 200 m; and the bit error rate: BER = 10${}^{-3}$. The transmitting range was chosen as an intermediate value in an outdoor environment, which refers to the 1 Mbps data rate of 802.11b technology. For the intention of simplification, we suppose that the transmitting range has a circular shape with the same transmitting power in each direction, see Figure 1. The bit error rate 10${}^{-3}$ is a typical value for an outdoor wireless environment.

**Figure 2:**
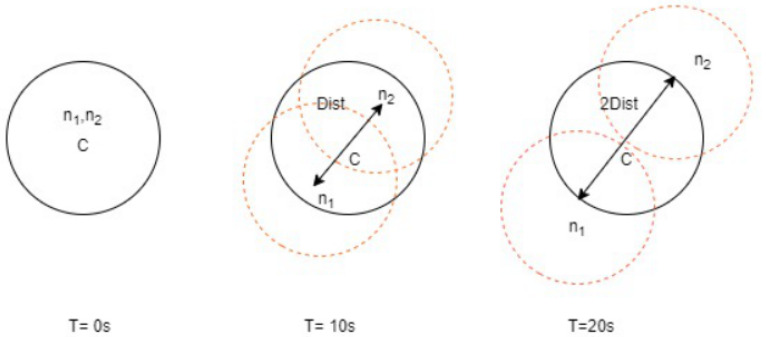
Distance measurements of mobile nodes.

Simultaneously moving straight from each other, we can calculate the maximum duration of communication according to Equation (1). Where dist is the maximum distance between nodes and s is the speed (speed) of their motion. In other words, it is the time when a node leaves the transmitting range of the second one and the communication will be ended. With respect to our initial premises, the value of the time interval t is according to Equation (2). This value suggests that the communication between two moving mobile nodes(n1 and n2), refer to Figure 2, will not be dismissed earlier than 20 s. We have assumed a time of 20 s and distance of 400 m. From a practical point of view, the time interval can be considered to be the initial value for the determination of the Hello interval and other routing protocol parameters. Figure 2 shows that mobile nodes are simultaneously moving directly away from each other:

(1) $$t=\frac{dist}{s}$$

(2) $$t=\frac{dist}{s}=\frac{400}{20}=20\;s$$

### 3.2. Network Design

**Figure 3:**
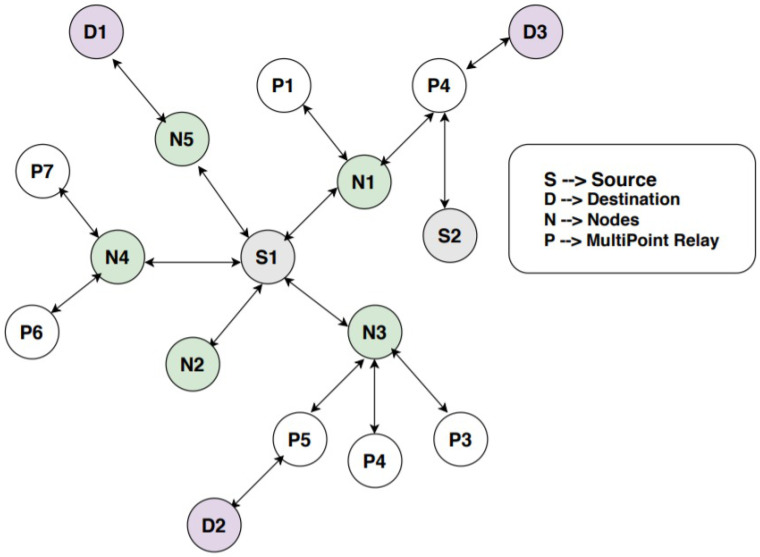
Network setup.

Let us assume deployment of mobile sensor nodes have multiple sensing diodes, a high-end micro controller and one or more transceiver as per the requirement of the application of Industry 4.0. As the size of the node is very small in terms of memory, processing unit and energy, 6LoWPAN is preferred for computation and execution process. The network is also assumed to work without a base station. All nodes are homogeneous in terms of functions. However, the head of the nodes requires control of the control interval of the messages. They are assigned weights with respect to their battery power, localization, mode of work (routers, wake or sleep), etc. Network setup can be observed from Figure 3, where it has 2 source nodes, 3 destination nodes, multiple relay nodes (one hop or two hop neighbor nodes) and multi point relay nodes. Coverage scalability is achieved in the graph G (with vertices V and edges E) with conditions:

(3) G={V,E}={Si}={Sm,Sd,Ss,Sr}

(*Si* = set of nodes n, *Sm* = set of multipoint nodes, *Ss* = set of source nodes, *Sr* = relay nodes, *Sd* = destination nodes).

In *Si*, there are four subgraphs composed of {Sm,Sd,Ss,Sr}, which helps in transmission and reception of data with the help of multipoint and relay nodes. The mathematical analysis is followed in the same approach as in [26,27]. It is performed to separate the nodes from each other in terms of their functions for smooth execution and to avoid unnecessary burden on the nodes. For optimized control intervals, it is necessary to divide the graph into subgraphs with particular functionality of the nodes. After division of nodes into different subsets with $(Sd=(V_{d},E_{d}$), $Ss=(V_{s},E_{s}))$ one to one nodes will be connected for the initiation of the session. Wireless links are of three types: (1) symmetric links where the protocol of transmission and reception of data is same, (2) asymmetric links where the protocol for transmission and reception is different, and (3) missing links, which indicates the missing routes due to early death of nodes or environmental conditions. The sensor nodes are elected by proactive routing protocol OLSR, but optimized control interval is implemented during the transmission of messages (proposed approach) and the shortest path based on cooperative communication as was proposed in [22] is selected to transmit the data. Afterward, a reactive method is followed to manage the ad hoc nature of nodes.

### 3.3. Nomination of Head on Basis of First Expectation Factor

Grouping of nodes is required because it reduces the total network costs by reducing severe flooding and evaluating a head of clusters. The head of the unit (responsible for handling of optimized control interval) is nominated according to the first defined expectation factor (FDEF). FDEF is the expected value of a random nominated head and generalization of weighted average, i.e., mathematical expectation. It can be implemented on both a finite and infinite number of nodes. However, here we are considering the ad hoc networks and number of nodes as finite; therefore, for finite number of nodes, the selection is made in the following manner:

Let $N_{i}$ be a random head with finite number of nodes $N_{1}$, $N_{2}$…, $N_{k}$ with probability $p_{1}$, $p_{2}$…, $p_{k}$, respectively. The expectation of N is defined as:

(4) $$E\left(N\right)=\sum_{i=1}^{k}nipi=ni1p1+ni2p2+nikpk$$

The sum of probabilities will be 1, the expected value will be computed with the help of assigned weights (according to remaining energy, optimized control interval and distance). The first expectation factor of the grouping head is based on the above three parameters. A head should have a minimum control interval, maximum remaining energy and less distance from source nodes and base station than other nodes, which will give this node more weightage for the grouping head than other nodes. If all outcomes are equal then the weighted average will become a simple average. If outcomes are not equal, then the average will be replaced by the weighted average (it shows that certain outcomes are most likely to happen than others). It is based on dice game theory.

### 3.4. Optimization of OLSR Parameters

Since more and intensive packet transmission leads to higher power consumption and quicker depletion of the battery, we focused on the attributes (distance, energy, hello interval) that are responsible for the reliable communication and the effects of their length on the amount of routing traffic. The hello interval parameter shows the time intermission among hello packets. These packets are required to maintain end-to- end communication between nodes.

Hello packets carry information of one and two hops neighbor’s details. The primary goal of our optimization is to reduce the amount of routing traffic and also minimize a time period. Time is measured as an interval between leaving the transmitting range of the current neighbor node and being registered in the neighbor’s list of another mobile node via reducing the delay of control messages. According to our assumptions, the value of 3 s should give the same effects with less control traffic because during *t* = 20 s, the mobile node sends six hello messages. The seventh hello message will be obtained by a new neighbor node in a new transmitting range. In the scenario shown in Figure 1, the time interval t is 1 s, which is an acceptable value. Following is the mathematical analysis of delay in transmitting the data as per the proposed approach.

1. End to End Stability: It shows the time period for which the packets are transmitted over all the channels. It is computed by Equation (5) where minimum transmission energy is used to transmit data from i to n in time t, i.e., $\min\_trans\_l^{i,n}{}_{t}$.

(5) $$E_{endtoend\_stability}\left(t\right)\;=\;\frac{1}{\sum_{n=1}^{hop}\frac{1}{\min\_trans\_l^{i,n}{}_{t}}}$$

2. Analysis of Delay: Proposed approach is useful for industry applications due to the reduction in delay and can be observed from the following analysis Equations (6)–(22). The description of the symbols used in the analysis of delay can be found in Table 2. The upper bound of the traffic is computed by Equation (6), where the total size of the data is δ tot.

(6) ε(t1,t1+tot)≤δtot+bukt_cap

The capacity of the channel is calculated with theShannon theorem, as given below.

(7) $$cap^{n}{}_{i}=B^{n}{}_{i}\log_{2}\left(1+snr_{i}^{n}\right)$$

Total capacity of all the links is:

(8) $$tot_{n}\_cap\left(t\right)=\sum_{i=1}^{n}cap_{n}$$

Service at that time for node n is given by Equation (9).

(9) $$tot\_ser_{n}\left(t\right)=\int_{t=0}^{tn}tot_{n}\_cap\left(t\right)$$

Effective bound or capacity of the function (ECF) is computed through Equations (10)–(12).

(10) $$\phi_{k}\left(-ℑ\right)=\lim_{x\to t}\frac{1}{tot}\log Ecap\left[e^{-ℑtot\_ser_{n}\left(t1\right)}\right]$$

(11) $$\beta_{k}\left(ℑ\right)=\frac{-\phi_{k}\left(-ℑ\right)}{ℑ}\forall ℑ\geq 1$$

For any channelm i.e., *k*, the ECF can be given as:

(12) $$\beta_{k}\left(ℑ\right)=\lim_{x\to t}\frac{1}{ℑt1}\log Ecap\left[e^{-ℑ\int_{t=0}^{n}tot\_ser_{n}\left(t1\right)}\right]$$

The process for *k* is given as below:

(13) $$\gamma_{i}\left(-\phi \right)=\lim_{x\to t}\frac{1}{t1}\log Ecap\left[e^{-ℑ\int_{t=0}^{n}tot\_ser_{n}\left(t1\right)}\right]$$

i.e.,

(14) $$a_{i}\left(\phi \right)=\frac{-\gamma_{i}\left(-\phi \right)}{\phi }\forall \phi \geq 0$$

(15) $$a_{i}\left(\phi \right)=\lim_{x\to t1}\frac{1}{ℑt1}\log Ecap\left[e^{-ℑ\int_{t1=0}^{n}tot\_ser_{n}\left(t1\right)}\right]\forall \phi \geq 0$$

Degree of the received process during that time interval of t is:

(16) $$\sigma_{k}\left(t1\right)=\sum_{ser=1}^{tot\_ser_{n}}\varepsilon^{ser}\left(t1,t1+tot\right)$$

ECF of the arrival traffic is:

(17) $$\chi_{i}\left(\phi \right)=\lim_{x\to t}\frac{1}{t1}\log Ecap\left[e^{-ℑ\sigma_{k}\left(t\right)}\right]$$

(18) $$\delta_{i}\left(\phi \right)=\frac{\chi_{i}\left(-\phi \right)}{\phi }\forall \phi \geq 0$$

The derivation of the received flow is:

(19) $$\delta_{i}\left(\phi \right)=\frac{\chi_{i}\left(e^{\phi }-1\right)}{\phi }$$

The difference between the capacity of the channel and received flow is determined by:

(20) $$Qe_{i}\left(t1\right)=\sigma_{k}\left(t\right)-\varphi_{i}\left(\phi \right)$$

Waiting time for the flow of the traffic is computed through Equation (21), and service delay can be observed via Equation (22).

(21) $$Ti_{i}^{Flow\_ser}\left(t1\right)=\frac{i}{Qe_{i}\left(t1\right)*\beta_{k}\left(\phi \right)}$$

(22) $$S_{delay}{}_{i=1}^{n}\left(t1\right)=\frac{1}{\sigma_{k}\left(t\right)*a_{i}\left(\phi \right)}$$

3. End to End Delay: For data transmission in industrial applications, it is required to compute the end to end delay while transmitting data from source to destination, and hence, for wireless communication, its cost can be observed from Equation (23).

(23) $$delay_{i}^{n}\left(t1\right)=\left[Ti_{i}^{Flow\_ser}\left(t1\right)+\right.\frac{Flow\_ser}{cap_{k}\left(t1\right)}*\frac{1}{1-err_{k}^{f}}$$

Switching delay for the route between source to destination is computed by Equation (24).

(24) $$delay_{path}^{ser\phi }\left(t1\right)=CS_{path}*delay^{ser\phi }$$

For hop length hop_len, the switching delay is Equation (25):

(25) $$endtoend\_delay_{path}\left(t1\right)=delay_{path}^{ser\phi }\left(t1\right)+A$$

(26) $$A=\sum_{i=1}^{hop\_len}delay_{i}^{n}\left(t1\right)$$

4. Bit Rate computation and end to end loss: In wireless communication, switching from one node to another node can lead to the loss of bits. Delay in this communication is computed via Equation (26). However, in terms of hop length, i.e., hop_len, the bit rate can be observed with Equations (27) and (28).

(27) $$pkt\_loss_{path}\left(t1\right)=1-\prod_{i=1}^{hop\_path}\left(1-pkt\_loss_{i}^{n}\right)$$

(28) $$avail_{path}^{bit\_rate}\left(t1\right)=\min\left(bit\_rate_{i}^{n}bit\_rate_{tot\_hop}^{n}\right)$$

5. Topology Control interval: The Topology Control (TC) interval specifies the time period between Topology Control messages. As per the algorithm, which is shown in Algorithm 1, TC messages are produced by MPR nodes to transmit connectivity or topology data. Control messages have the topology table for each guest. This information is utilized for forming an updated routing table. Agreeing to our assumptions, the TC interval should be twice the hello interval, which is 6 s. This value was set in terms of the computations similar to the hello interval. During t = 20 s, the mobile node sends 3 TC messages. The fourth TC message will be picked up by a new neighbor node in a new transmitting range.

6. Neighbor’s holding time interval: This parameter determines the link expiry time. This is typically set to three times the value of the hello interval. With each hello message arriving, the link expiry timer is reset. In the case of no reception of in a particular duration of time, the link is assigned an ID, i.e., lost. In the case of failure of all links to a neighbor node, the neighbor is declared to be out of reach.

7. Topology time interval: This parameter defines the expiry time for values stored in the topology table. This is typically set to three times the value of the default TC interval.

## 4. Result Analysis and Discussions

The procreative results for OLSR expansions were investigated on the justification of delay parameters, reloading, hello message, number of topology and expected performance control messages. With its traditional methods rule, the upgraded OLSR was reviewed in isolation. The simulations were carried out in MATLAB. The results of the modernization are still seen as diagrams, which mostly contradict the summary of upgraded and traditional OLSR. Improved and optimized OLSR has shown its improvement over other routing techniques, i.e., DSDV, AOMDV, hybrid routing and OLSR.

### 4.1. Escalate OLSR: Delay

**Figure 4:**
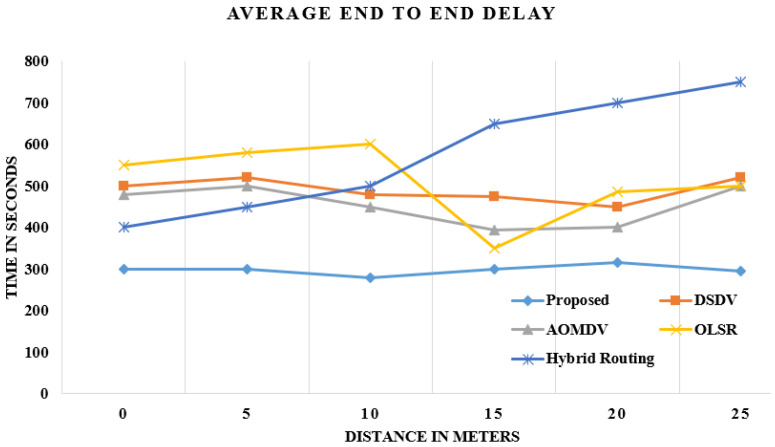
Comparison of delay in data reception.

The DELAY is the entire final delay of large proportions of bundles that are gathered and sent to appropriate levels via Wireless LAN MACs for all wired WLAN nodes in the mechanism. The strengthened 70-node OLSR highlights literally a nearly approximate equal delay. The optimized OLSR has shown less delay than the comparative algorithms. In each step, the grouping in, i.e., head selection, has improved OLSR, which selects a cluster face and significantly reduces origin time to the overarching goal, as shown in Figure 4.

In every round, the head of the cluster will be nominated with probability p. Every node is approximately equal in terms of functions as explained in the above subsection of network design. The strengthening approach of optimized OLSR reduces the amount of delay.

### 4.2. Escalate OLSR: Load

**Figure 5:**
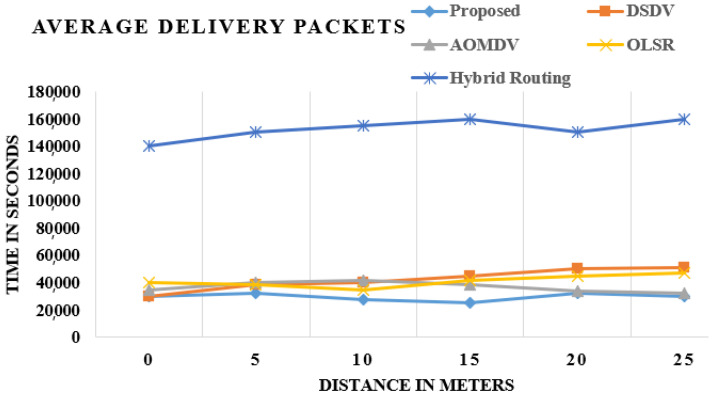
Comparison of packet delivery.

Load establishes the absolute load made available by the higher layers in a mechanism for wireless LAN layers. Figure 5 shows the standard practice and augmented OLSR load. In way of comparison to OLSR, DSDV, AOMDV and Hybrid routing, the upgraded OLSR seems to have the gradual decline in load and default. The load is the trustworthiness variable. As a proportion of the unwavering consistency determinant for the two adaptations, the robustness of accelerated OLSR is and has always been similar, as shown in Figure 5.

These charts demonstrate the correlation of existing and proposed strategies, and the outcome shows that average packet delivery is the most extreme in the proposed procedure in contrast with the current methods.

### 4.3. Sent Hello Messages

OLSR has dynamically picked MPR center points, which moreover shows that the improved OLSR has a large number of hello message exchanges than traditional OLSR. The aim behind welcome messages is to identify the status of the associations, for instance, the interface is SYN (synchronization), ASYN (asynchronization) or LOST (missed), and to pick the MPR’s center points for the framework. The optimized OLSR shows the high approximation of the hello messages exchange compared to the default OLSR, AOMDV, hybrid routing and DSDV.

**Figure 6:**
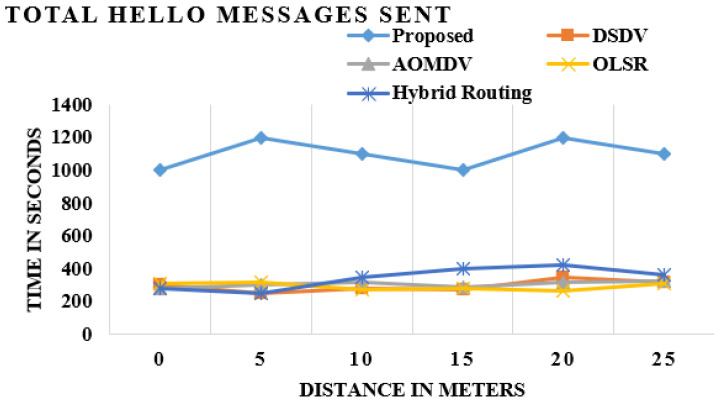
Comparison of hello message sent.

The certifiable sentiment of associations foresees the packages sent to the missing links center. The packets are simply guided to the SYN center, which is equipped for ending up being the bundle head. It overhauls the framework execution, as shown in Figure 6.

### 4.4. Sent Message Control Topology

**Figure 7:**
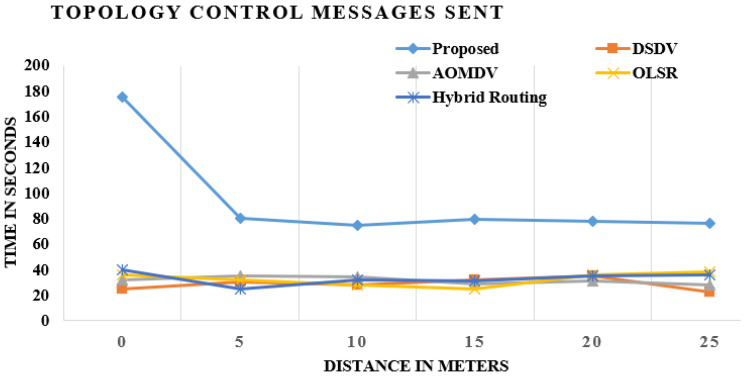
Comparison of existing and proposed techniques in transmitted control messages.

The control topology message illustrates all messages sent to TC with each node within and without the structure. The MPR node distributes topology control messages that incorporate topology in the longest period of time and consistency. The transition in topology depends on the voracious measurement. The more the structure designates the MPR nodes, the greater the chance of topology control notices. Figure 7 exemplifies OLSR TC communication networks standard and modified OLSR with AOMDV, DSDV and hybrid routing. TC’s observations in the upgraded OLSR are much stronger than in existing approaches, as shown Figure 7.

These diagrams demonstrate the correlation of existing methods and the proposed system, and the outcome demonstrates that the topology control message sent is the greatest in the proposed strategy when contrasted with the existing procedure.

### 4.5. Throughput

**Figure 8:**
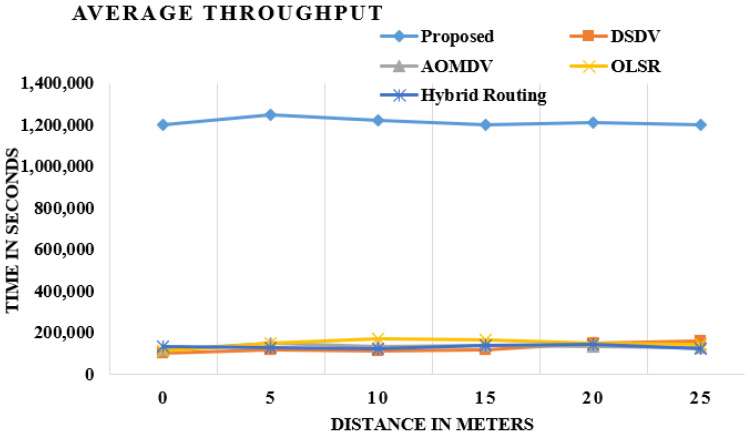
Comparison of existing and proposed techniques in average throughput.

The good performance is defined in terms of the remaining balance of bits sent to the overhead layer nodes from the WiFi. Figure 8 shows OLSR performance requirements and improvements [18]. The performance is good for smarter OLSRs, as OLSR strengthens its MPR, Hello and TC nodes components. The cluster’s head is known as the forwarder node and the group of origin of many of these nodes. In this way, it turns out to be simple for the forwarder packet to advance the bundle to a destination node without looking through the entire system. The upgraded OLSR has demonstrated a lot higher throughput than the customary OLSR, AODV, DSDV and hybrid routing, as shown in Figure 8.

These figures demonstrate the examination of the existing system and proposed procedure, and the outcome demonstrates that the normal throughput change is most extreme in the proposed strategy when contrasted with the existing method.

The overall performance of the proposed work in the throughput, transmitted control messages and hello messages, end-to-end delay and average delivery of packets improved by 70%, 40%, 50%, 32% and 60%, respectively, over the DSDV, AOMDV, OLSR and Hybrid Routing.

## 5. Conclusions

In this research work, the new enhanced version of the original OLSR protocol is formulated and demonstrated by changing all the control intermissions in OLSR and by means of the best value for all intermissions, which is crucial for providing optimal outcomes for the network. The proposed enhanced OLSR scheme embedded the clustering phenomena, which is a prominent approach to enhance the outcomes of a routing scheme. It is due to the fact that it lowers the complete network cost by reducing flooding and further selecting a cluster head on the basis of a significant probability factor. The implementation and analysis of the routing protocol is carried out in MATLAB. The performance of the proposed scheme is approximately three times better than AOMDV, DSDV, hybrid routing and OLSR. The obtained results concluded that the improved OLSR version yields better execution results over the existing schemes while dealing with various parameters, thereby making the network much more effective and reliable for Industry 4.0. In IoT, heterogeneous devices are becoming connected and giving rise to new constraints as per the increased demand of the users. A modification of the proposed method will be required for on-demand services. In future, the federated learning will be used for Smart Grids.

## Figures and Tables

**Table 1:** Symbols used in mathematical analysis.

Notation	Meaning
s	Speed
r	radius
BER	bit error rate
t	time
dist	distance
G	graph
V	vertices
E	edges
*Si*	set of nodes
*Sm*	set of multipoint nodes
*Ss*	set of source nodes
*Sr*	set of relay nodes
*Sd*	set of destination nodes
p	probability
Ni	nodes
Tc	topology control

**Table 2:** Meaning of symbols used in equations for delay in traffic flow.

Meaning	Symbol
Upper Bound of the Traffic	ε(t1,t1+tot)
Channel Capacity	cap${}^{n}{}_{i}$
Total Capacity of all the links	tot${}_{n}\_cap\left(t\right)$
Service for node n at time t	tot$\_ser_{n}\left(t\right)$
Effective Capacity	$\beta_{k}\left(ℑ\right)$
Effective Bound	$\phi_{k}\left(-ℑ\right)$
Channel (*k*) Processing	$\beta_{k}\left(ℑ\right)$
Degree of the processes received at time interval *t*	$\sigma_{k}\left(t1\right)$
Effective Capacity Function of Arrival Traffic	$\chi_{i}\left(\phi \right)$
Received Flow	$\delta_{i}\left(\phi \right)$
Difference between capacity of the channel and received flow	$Qe_{i}\left(t1\right)$
Waiting time for the flow of traffic	Ti${}_{i}^{Flow\_ser}$ (t1)
Service Delay for the traffic	S${}_{delay}{}_{i=1}^{n}\left(t1\right)$
End to end delay	delay${}_{i}^{n}\left(t1\right)$
Switching Delay between Source to Destination	delay${}_{path}^{ser\phi }$ (t1)
Delay between hops	endtoend$\_delay_{path}\left(t1\right)$
Bit loss due to switching	pkt$\_loss_{path}\left(t1\right)$
Bit Rate	avail${}_{path}^{bit\_rate}\left(t1\right)$

## Data Availability

Not applicable.
